# Highly-twisted states of light from a high quality factor photonic crystal ring

**DOI:** 10.1038/s41467-023-36589-8

**Published:** 2023-02-27

**Authors:** Xiyuan Lu, Mingkang Wang, Feng Zhou, Mikkel Heuck, Wenqi Zhu, Vladimir A. Aksyuk, Dirk R. Englund, Kartik Srinivasan

**Affiliations:** 1 Microsystems and Nanotechnology Division, Physical Measurement Laboratory, National Institute of Standards and Technology, Gaithersburg, MD 20899 USA; 2 Joint Quantum Institute, NIST/University of Maryland, College Park, MD 20742 USA; 3 Department of Chemistry and Biochemistry, University of Maryland, College Park, MD 20742 USA; 4 Department of Electrical and Photonics Engineering, Technical University of Denmark, Lyngby, 2800 Kgs. Denmark; 5 Department of Electrical Engineering and Computer Science, Massachusetts Institute of Technology, Cambridge, MA 02139 USA

**Keywords:** Nanophotonics and plasmonics, Microresonators

## Abstract

Twisted light with orbital angular momentum (OAM) has been extensively studied for applications in quantum and classical communications, microscopy, and optical micromanipulation. Ejecting high angular momentum states of a whispering gallery mode (WGM) microresonator through a grating-assisted mechanism provides a scalable, chip-integrated solution for OAM generation. However, demonstrated OAM microresonators have exhibited a much lower quality factor (*Q*) than conventional WGM resonators (by >100×), and an understanding of the limits on *Q* has been lacking. This is crucial given the importance of *Q* in enhancing light-matter interactions. Moreover, though high-OAM states are often desirable, the limits on what is achievable in a microresonator are not well understood. Here, we provide insight on these two questions, through understanding OAM from the perspective of mode coupling in a photonic crystal ring and linking it to coherent backscattering between counter-propagating WGMs. In addition to demonstrating high-*Q* (10^5^ to 10^6^), a high estimated upper bound on OAM ejection efficiency (up to 90%), and high-OAM number (up to *l* = 60), our empirical model is supported by experiments and provides a quantitative explanation for the behavior of *Q* and the upper bound of OAM ejection efficiency with *l*. The state-of-the-art performance and understanding of microresonator OAM generation opens opportunities for OAM applications using chip-integrated technologies.

## Abstract (web summary)

Twisted light in the form of orbital angular momentum (OAM) can provide an additional spatial dimension for information transmission. Here, the authors demonstrate a prescription for OAM generation using photonic crystal ring resonators, in which high cavity quality factors (up to 10^6^) are retained.

## Introduction

Light with orbital angular momentum (OAM)^1,2^, previously known as helically phased light^3,4^, has been of long-standing interest. As an intrinsic property of photons, OAM with quantum number *l* provides an additional dimension to encode information^5^. This extra information capacity has been harnessed in holography^6–8^, multiplexed communications^9–12^, quantum entanglement^13–15^, and cryptography^16,17^. After Allen et al. pointed out that OAM is a natural property of all helically phased beams^3^, it has been routinely generated in free space based on traditional helical beam generation methods^3,18,19^. Recently, thin film metasurfaces have been used as a single-layer alternative to more traditional multi-level phase plates^20–22^, and OAM light with *l* up to 276 has been shown^23^. In addition, spiral phase mirrors have been used to generate photons carrying OAM with ∣*l*∣ > 10,000, with its OAM (±*l*) entangled with another photon’s horizontal/vertical (H/V) polarization^24^.

On-chip OAM generation using integrated photonics^25^ can advance more widespread use of OAM functionalities, and one major approach in this regard is through whispering gallery mode (WGM) microresonators^26^. The WGMs in such resonators are bound modes that naturally support high angular momentum, and OAM-carrying states can be realized if a suitable means to eject such WGMs into free space is incorporated, e.g., through a grating inscribed on the resonator^27^. For a WGM with azimuthal order *m*, a grating with *N* periods around the resonator circumference will eject light carrying OAM with *l* = *m*−*N*.

The WGM approach is distinguished by the ability to simultaneously enhance light–matter interactions through the microresonator’s high-quality factor (*Q*) and small mode volume (*V*)^28^. This has been used, for example, in OAM semiconductor microlasers^29,30^ and in OAM single-photon sources based on the Purcell-enhanced emission of a single quantum emitter by the WGM^31^. To maximize the microresonator’s ability to enhance interactions while ejecting light into an OAM state, its high *Q* should be retained even in the presence of the ejection grating, with the degradation in *Q* relative to a conventional resonator (no grating) being exclusively due to the new coupling channel into the free-space OAM mode. This behavior should hold for a wide range of *l*, to fully enable the spatial multiplexing at the heart of OAM’s potential in quantum and classical communications. However, existing demonstrations of OAM-generating microresonators have been limited to *Q* ≈ 10^3^ so far^27,31,32^, and have focused on relatively low-*l* OAM states. We believe these two limits for OAM in WGMs are in large part due to the lack of quantitative understanding of the relationship between *Q* and OAM ejection efficiency and *l*. Without such an understanding, the full potential of such devices has remained unexplored.

Improving *Q* in OAM-generating resonators has numerous implications. For example, in single quantum emitter systems, higher *Q*s would produce stronger Purcell enhancement to improve the indistinguishability and spontaneous emission coupling fraction of OAM single photons, with the further possibility of entering the non-perturbative strong coupling regime of cavity QED^33^. A second example is the spatiotemporal shaping of light^34^, where the ability to control both the spatial and temporal degrees of freedom of light is of both fundamental interest and can lead to new abilities for optical manipulation^35^. Recently, dynamic spatiotemporal control has been explored in the context of the coherent addition of optical frequency comb components that carry different amounts of OAM^36^. Recent advances in frequency comb generation through nonlinear wave mixing in microresonators^37^ suggest its potential in such research, but the limited *Q*s of OAM microresonators and the lack of understanding of these limits have prevented any serious investigation of such opportunities.

Here, we demonstrate chip-integrated, high-*Q* (10^5^–10^6^) microresonators that generate high-*l* OAM states (up to *l* = 60) with a high estimated upper bound of OAM ejection efficiency (up to 90%). We also provide a model that predicts the OAM ejection efficiency and microresonator’s total dissipation rate and scaling with *l*. We do so by considering how OAM generation is one manifestation of grating-assisted coupling in a microresonator. In particular, we establish a connection between OAM ejection and mode-selective backscattering, known as selective mode splitting (SMS)^38^, and show how measurements of SMS devices enable quantitative predictions of OAM behavior that are well-matched by experiments. Along with performance that dramatically exceeds previous studies in terms of *Q* and accessible OAM states, our work provides a foundation for further development of OAM generation, particularly in the context of nonlinear and quantum light sources.

## Results

### Principle Idea

**Fig. 1:**
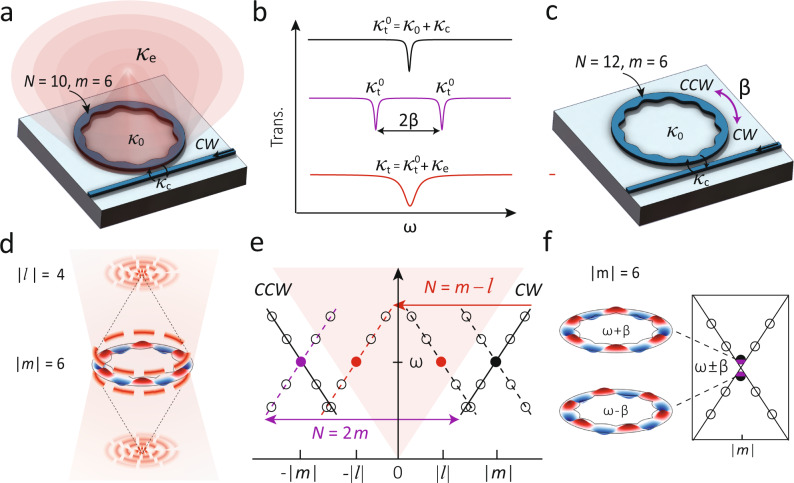
Linking orbital angular momentum (OAM) emission with selective mode splitting (SMS) in a photonic crystal microring (PhCR). **a–c** Schematic of two PhCRs with *N* = 10 (**a**) and *N* = 12 (**c**), where *N* is the number of modulation periods in a round trip. Their transmission spectra are illustrated in (**b**). Here, we focus on a whispering gallery mode (WGM) with an azimuthal mode number *m* = 6. Similar to a conventional (unmodulated) microring, this WGM has an intrinsic loss rate of *κ*_0_ (from incoherent scattering and absorption) and is evanescently coupled by a waveguide with a coupling rate *κ*_c_. The laser injected from the right side of the waveguide excites the clockwise WGM. In **a**, the PhCR with *N* = *m* + 4 = 10 leads to OAM light emission, at an ejection rate *κ*_e_, and carrying angular momentum of *l* = −4. In **b**, the PhCR with *N* = 12 leads to a coupling of clockwise and counter-clockwise WGMs with *m* = ±6, at a coupling rate *β*. **d** In the OAM device, the grating with *N* = 10 ejects the clockwise mode with *m* = 6 into a free-space OAM mode carrying a momentum of *l* = −4 at a rate of *κ*_e_. The OAM emission leads to a broadening of the cavity linewidth (${\kappa }_{{{{{{{{\rm{t}}}}}}}}}={\kappa }_{{{{{{{{\rm{t}}}}}}}}}^{0}+{\kappa }_{{{{{{{{\rm{e}}}}}}}}}$). When the WGM is a standing wave with *m* = ±6, or there is a reflection in the microring or chip facet (e.g., reflecting *m* = 6 to *m* = −6), the observed OAM mode has *l* = ±4, which is manifested in its intensity profile exhibiting 4 pairs of anti-nodes. **e** Schematic band diagram for OAM and SMS. The purple double-arrow indicates the coupling between the clockwise and counter-clockwise traveling wave WGMs. The red single-arrow indicates the one-way ejection of light from the clockwise mode to the free space OAM mode. The OAM emission is symmetric to clockwise and counter-clockwise modes because the grating is static. **f** In the SMS device, the grating with *N* = 12 couples the clockwise and counter-clockwise modes with *m* = ±6 to each other without introducing excess loss (that is, ${\kappa }_{{{{{{{{\rm{t}}}}}}}}}^{0}={\kappa }_{{{{{{{{\rm{0}}}}}}}}}+{\kappa }_{{{{{{{{\rm{c}}}}}}}}}$), but introduces a mode splitting of 2*β* between the two renormalized standing-wave modes.

OAM ejection from a WGM is well-understood at a qualitative level, based on the basic angular momentum conservation criterion between the initial WGM with angular momentum *m*, the imprinted grating with *N* periods along the ring circumference, and the resulting ejected OAM state with *l* = *m*−*N*^27^, as illustrated in Fig. 1a, d. However, the key missing point is an understanding of the strength of the coupling from the WGM to the free-space OAM mode, which we quantify by a rate *κ*_e_. This coupling leads to additional broadening of the total cavity linewidth, given by ${\kappa }_{{{{{{{{\rm{t}}}}}}}}}={\kappa }_{{{{{{{{\rm{t}}}}}}}}}^{0}+{\kappa }_{{{{{{{{\rm{e}}}}}}}}}$, where ${\kappa }_{{{{{{{{\rm{t}}}}}}}}}^{0}$ includes the WGM intrinsic loss rate *κ*_0_ and waveguide coupling rate *κ*_c_, which is well-understood in conventional microrings. Such broadening is illustrated in Fig. 1b. On the other hand, the interaction rate between two counter-propagating WGMs mediated by an imprinted grating, termed selective mode splitting (SMS), is well-understood at a quantitative level^38^. Here, we use a photonic crystal ring (PhCR) as an example, as shown in Fig. 1c. The inside radius of the PhCR is modulated as ${R}_{{{{{{{{\rm{in}}}}}}}}}={R}_{{{{{{{{\rm{in}}}}}}}}}^{0}+A\cos$ (*Nϕ*), where ${R}_{{{{{{{{\rm{in}}}}}}}}}^{0}$ is the average inside radius, *A* is the modulation amplitude, *N* is the number of periods of the grating, and *ϕ* is the azimuthal angle. Each WGM in the PhCR is characterized by an azimuthal mode number *m*, representing its angular momentum, that is, the number of electric field oscillations around the device perimeter within one round trip. When *m* = *N*/2, the clockwise and counterclockwise WGMs are coupled by the photonic crystal grating. This coupling renormalizes two propagating modes into two standing-wave modes that see a narrower and a wider ring on average, and therefore have a smaller and larger resonance wavelength, or equivalently, a higher and lower center resonance frequency (*ω*_±_ = *ω*_0_ ± *β*), respectively, as illustrated in Fig. 1f, where *ω*_0_ is the uncoupled (clockwise or counter-clockwise propagating) mode frequency. The coupling rate *β* is simply given by *β* = *gA*, where *A* is the modulation amplitude of the inside radius and *g* = ∂*ω*/∂*R*_in_ at ${R}_{{{{{{{{\rm{in}}}}}}}}}={R}_{{{{{{{{\rm{in}}}}}}}}}^{0}$, with *ω* the angular frequency of the WGM. We note that *g* can be intuitively understood as the geometric dispersion with respect to the inside radius of an unmodulated ring. It is also equivalent to the per photon force (divided by *ℏ*) on the inside boundary of an unmodulated ring. Importantly, SMS WGMs remain high-*Q*^38–40^ (${Q}_{{{{{{{{\rm{t}}}}}}}}}^{0}=\omega /{\kappa }_{{{{{{{{\rm{t}}}}}}}}}^{0}$), with ${\kappa }_{{{{{{{{\rm{t}}}}}}}}}^{0}$ remaining the same as in a conventional microring, that is, ${\kappa }_{{{{{{{{\rm{t}}}}}}}}}^{0}={\kappa }_{{{{{{{{\rm{0}}}}}}}}}+{\kappa }_{{{{{{{{\rm{c}}}}}}}}}$, as illustrated in Fig. 1b.

The contrast of the poor understanding of *κ*_e_ in OAM with the clear understanding of *β* in SMS is striking when we consider the similarity of these two systems, namely, that the number of periods in the grating (*N*) is the only difference in device geometry, with *N* = *m*−*l* for the OAM light carrying *l* momentum, and *N* = 2*m* for SMS. The geometries of the OAM and SMS devices are illustrated in Fig. 1a, c, with their momentum–frequency diagrams shown in Fig. 1e. The OAM mode is ejected from the device and cannot interact with the WGM mode after emission, as shown in Fig. 1a, while clockwise and counter-clockwise WGMs can scatter back and forth, as shown in Fig. 1c. In the band diagram shown in Fig. 1e, the OAM emission is illustrated by a red arrow and the SMS coupling is illustrated by a purple double-ended arrow, assuming the waveguide initially couples light into the WGM in the clockwise direction. Figure 1b shows the expected transmission spectra of the control device (without modulation), the SMS case, and the OAM case. Compared to the control device, the SMS device shows a frequency splitting but no linewidth broadening, while the OAM device shows a linewidth broadening but no frequency splitting.

From coupled-mode equations for OAM and SMS (see the Supplementary Information for details), we propose a link between OAM and SMS given by

1 $${\kappa }_{{{{{{{{\rm{e}}}}}}}}}={q}_{0}\frac{2\beta }{\sqrt{{F}_{{{{{{{{\rm{t}}}}}}}}}/(2\pi )}}\cos (\theta ).$$

*κ*_e_ and *β* have the same units (both are rates), while all other parameters here are unitless. *q*_0_ is a constant, and *F*_t_ is the cavity mode finesse given by *F*_t_ = *Q*_t_/*m* = *ω*/(*mκ*_*t*_), where *Q*_t_, *ω*, and *κ*_*t*_ are the total optical quality factor, cavity resonance angular frequency, and total cavity linewidth, respectively, of the corresponding WGM mode with an angular momentum of *m* in the microring and angular momentum of *l* = *m*−*N* in the OAM emission. *θ* = (*l*/*m*)(*π*/2) represents the nominal twisted angle of the ejected OAM modes with respect to the vertical direction. Writing *κ*_t_ in terms of its original value with no OAM emission (${\kappa }_{{{{{{{{\rm{t}}}}}}}}}^{0}$) and OAM emission rate (*κ*_e_), we get:

2 $${\kappa }_{{{{{{{{\rm{t}}}}}}}}}={\kappa }_{{{{{{{{\rm{t}}}}}}}}}^{0}+2q\sqrt{{\kappa }_{{{{{{{{\rm{t}}}}}}}}}}.$$

${\kappa }_{{\rm {t}}}^{0}$ includes the cavity intrinsic loss rate and waveguide–ring coupling rate, so that ${\kappa }_{{{{{{{{\rm{t}}}}}}}}}^{0}={\kappa }_{0}+{\kappa }_{{{{{{{{\rm{c}}}}}}}}}$. *q* is related to *κ*_e_ and *κ*_t_ by ${\kappa }_{{{{{{{{\rm{e}}}}}}}}}=2q\sqrt{{\kappa }_{{{{{{{{\rm{t}}}}}}}}}}$, with $q={q}_{0}\beta \sqrt{m/\nu }\cos (\theta )$ (see Eq. (1)). Equation (2) is a quadratic function of $\sqrt{{\kappa }_{{{{{{{{\rm{t}}}}}}}}}}$, and its solution is given by

3 $$\sqrt{{\kappa }_{{{{{{{{\rm{t}}}}}}}}}}=q+\sqrt{{q}^{2}+{\kappa }_{{{{{{{{\rm{t}}}}}}}}}^{0}},$$

where the other solution is negative and discarded.

From these simple equations, we can make a few initial observations. In the SMS case, where *l* = −*m* (*N* = 2*m*), the cosine term vanishes, so that *q* and *κ*_e_ are zero. This is consistent with previous observations^38,40^ where *κ*_t_ is barely affected by the grating modulation as long as *N* = 2*m*. When *l* = 0, i.e., *N* = *m*, corresponding topologically to the *LG*_01_ mode in the Laguerre–Gaussian basis of modes (*LG*_*lp*_, where *l* represents the angular momentum number and *p* represents the radial momentum number), the cosine term is equal to one. In this case, when *β* and *κ*_e_ are small, the cavity linewidth asymptotically approaches that of the unmodulated microring (${\kappa }_{{{{{{{{\rm{t}}}}}}}}}\, \approx \,{\kappa }_{{{{{{{{\rm{t}}}}}}}}}^{0}$). When *κ*_e_ is large compared to ${\kappa }_{{\rm {t}}}^{0}$, the OAM ejection channel is the dominant cavity loss channel (*κ*_t_ ≈ *κ*_e_). Finally, we posit that ${\kappa }_{{{{{{{{\rm{e}}}}}}}}}\propto \cos (\theta )$, i.e, the OAM ejection rate is linearly proportional to the momentum projected in the vertical direction after the grating’s momentum is exerted on the WGM. This assumption requires experimental verification.

### Experimental examination from SMS to OAM

**Fig. 2:**
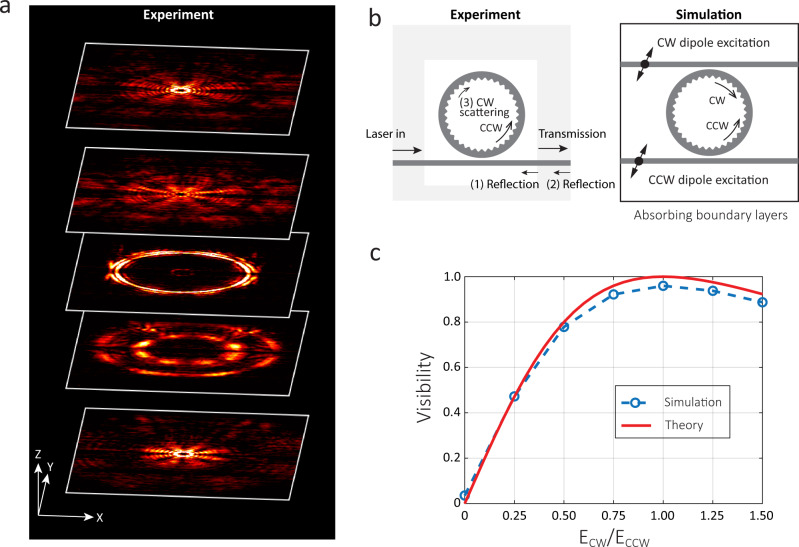
Representative OAM emission and self-interference. **a** OAM emission profile for ∣*l*∣ = 4. Experimentally measured mid-field and far-field infrared images of the *m* = 165 mode on a device with *N* = 169. These five images correspond to different heights of focal planes of the imaging system at approximately {70, 30, 0, −30, −70} μm with respect to the microring. The square boxes correspond to 80 μm × 100 μm on the *x*–*y* plane. The *x*–*y*–*z* arrows also serve as scale bars, whose lengths correspond to {20, 50, 20} μm, respectively. **b** Experimental and simulation schematics showing that in-plane reflection can lead to the observed 2∣*l*∣ beating patterns. **c** The simulated visibility $(|E{|}_{\max }^{2}-|E{|}_{\min }^{2})/(|E{|}_{\max }^{2}+|E{|}_{\min }^{2})$ (blue) agrees with a simple equation modeling the reflection by two CW and CCW dipoles (∣*E*_CW_cos(*lϕ*) + *E*_CCW_cos(−*lϕ*)∣^2^) (red).

We design and fabricate SMS and OAM devices in stoichiometric silicon nitride following the prescription of the previous section, with details provided in the Methods. Representative experimentally measured infrared images of the light ejected from one OAM device at various *z* (vertical) planes are shown in Fig. 2a. This device has *m* = 165 and *N* = 169, and the infrared images show OAM light with ∣*l*∣ = 4.

The OAM emission direction here is mainly vertical with a divergence angle, but also has a radial contribution with a Bessel pattern, as shown in Fig. 2a. This Bessel pattern is known to be generated when a plane wave passes through a ring slit and is focused by a lens^41^. In our case, the ring slit is naturally there by the WGMs within the microring, and the focusing is provided by the transferring of the angular momentum of the WGM to the OAM light by the inner sidewall grating.

A key feature of the OAM beam is the helical property carrying its orbital angular momentum. In a microring, it is represented by the angular momentum number *l*, assuming *E*(*r*, *z*) ≈ *E*_0_(*r*, *z*)e^*ilϕ*^e^*ikz*^. This simplified representation is made possible because of the rotational symmetry of a microring, and in a more complicated case (for example, in a racetrack ring), this simple equation will not hold, though a generalized *l* can still be used to describe the topological behavior. This helical feature has been confirmed by interference with left-/right-hand polarized beams^27^ or self-interference with an offset^29^. In this work, we observe this feature by self-interference in the microring, which results in a 2∣*l*∣ intensity beating pattern. For example, in Fig. 2a, we observed the interference patterns with 4 × 2 nodes in both the mid-field and far-field that are from the interference of OAM light with *l* = −4 and *l* = 4. These interference patterns rotate slowly when propagating in the z direction, likely due to the difference (in either propagation speed or spatial pattern) between the emitted ±*l* light. Going forward, we use such images to identify the *l* number for each OAM state, while also considering *Q* through transmission spectroscopy.

We conclude that such an intensity interference pattern is mainly attributable to the in-plane reflection channels from (1) the chip facets, (2) backscattering within the microring, and (3) the air/oxide cladding interface, as shown in the left schematic of Fig. 2b. The ending result of these three channels are equivalent and can be simulated by the structure shown in the right schematic of Fig. 2b. The simulated radiation pattern has visibility in intensity with 2*l* beating nodes, where the visibility is calculated by $(|E{|}_{\max }^{2}-|E{|}_{\min }^{2})/(|E{|}_{\max }^{2}+|E{|}_{\min }^{2})$, with ∣*E*∣^2^ extracted from a full 3D finite-difference time-domain simulation. As shown in Fig. 2c, the simulated results agree with a simple theoretical prediction of ∣*E*_CW_cos(*lϕ*) + *E*_CCW_cos(−*lϕ*)∣^2^. The visibility vanishes when there is no reflection (only CCW dipole, no CW dipole), and equals unity when *E*_CW_/*E*_CCW_ = 1 (CW and CCW dipoles have the same strength). We note that the out-of-plane reflections are not mainly responsible for creating such patterns in the current case; see the simulation results in Supplementary Fig. S2.

**Fig. 3:**
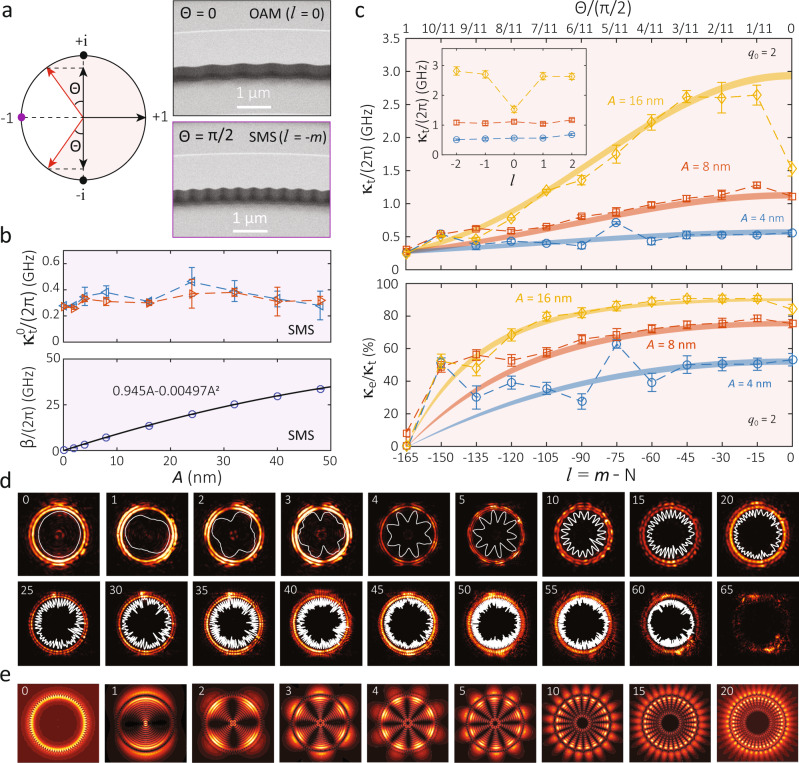
Coherent emission of OAM light from high-*Q* whispering gallery modes. **a** (Left) Illustration of the angle *θ*, which appears in Eq. (1) in describing the OAM ejection rate *κ*_e_, and (right) scanning electron microscope images of devices with *θ* = 0 (OAM) and *θ* = *π*/2 (SMS), respectively. The top image shows ≈5 periods of a PhCR for OAM (*N* = *m* = 165), while the bottom image shows ≈10 periods of a PhCR for SMS (*N* = 2*m* = 2 × 165). Both devices have *A* = 32 nm. **b** In the SMS case, ${\kappa }_{{{{{{{{\rm{t}}}}}}}}}^{0}$ of the two standing wave modes are not affected by increasing *A*, as shown in the top panel. The error bars are 95% confidence intervals of the nonlinear fits to the cavity transmission data (see Supplementary Fig. S3 for the details). The mode splitting (*β*) is nearly linearly dependent on *A* for the targeted WGM mode, whose experimental data and fit are shown in the bottom panel. The error bars are ≈0.3 GHz and are within the data symbols. **c** Plot of the OAM cavity linewidth (*κ*_t_) and estimated OAM ejection efficiency (*κ*_e_/*κ*_t_) in the top and bottom graphs, respectively, as a function of *l* = *m*−*N* on the bottom *x*-axis and *θ*/(*π*/2) = ∣*l*/*m*∣ on the top *x*-axis. We investigate three sets of devices having different modulation amplitudes of *A* = {4, 8, 16} nm, with *N* varying from 2*m* to *m* (i.e., transiting from the SMS regime to the OAM regime). We focus on a specific WGM at *ω*_0_ with mode number *m*_0_ in each set of devices. The error bars represent the 95% confidence intervals from nonlinear least-squares fits to the transmission data. The translucent shaded curves are from theoretical estimates predicted by Eqs. (1)–(3) with *q*_0_ = 2, with all other parameters taken from measurements of OAM and SMS devices. The width of each shaded curve originates from the combined range of six fitted ${\kappa }_{{\rm {t}}}^{0}$ values from three SMS devices at *l* = −165. The top inset shows a region of a discrepancy between the experiments and the model, near *l* = 0. The coupling waveguide in use has a nominal width of 750 nm and a microring-waveguide gap of 500 nm. **d** Infrared images near the surface of the OAM microrings for different ∣*l*∣ values, with each displaying 2∣*l*∣ anti-nodes. The angular intensity distribution is re-scaled and plotted in white to guide viewing. **e** Predicted patterns from three-dimensional finite-difference time-domain simulations using dipole excitation. While the OAM states are the same as in the experiments, a smaller device size (radius of 12 μm) and symmetric air cladding are used to keep the simulation size tractable.

We next consider the close connection between SMS and OAM devices, with representative devices shown in Fig. 3a. The length of a modulation period, given by 2*πR*/*N*, is twice as long in this OAM device (*N* = *m*, i.e., *l* = 0) as in the SMS device (*N* = 2*m*), but all other parameters are kept the same. We fabricate a series of devices for SMS and OAM, varying *N* while keeping the device geometry otherwise fixed. By studying modes of the same azimuthal order *m* and similar resonance frequency *ω*, we endeavor to limit the impact of any systematic variation in intrinsic and coupling *Q* (e.g., with frequency, ring width, thickness, refractive index, etc.), enabling us to focus on how *κ*_t_ and *κ*_e_ vary with *l* = *m*−*N*.

The SMS results are summarized in Fig. 3b, and are consistent with previous reports^38–40^: the total cavity linewidths (${\kappa }_{{{{{{{{\rm{t}}}}}}}}}^{0}$) see no change to within measurement uncertainty when *A* increases, and the mode splitting (2*β*) is essentially linearly dependent on *A* when the splitting is >10 × smaller than the free spectral range (≈1 THz in these devices). The error bars represent 95% confidence intervals from nonlinear least-squares fits to the SMS transmission data (see Supplementary Information). The measured ${\kappa }_{{{{{{{{\rm{t}}}}}}}}}^{0}/(2\pi ) \,\approx\, 0.3$ GHz corresponds to a ${Q}_{{\rm {t}}}^{0} \,\approx\, 6.4 \times 1{0}^{5}$ at 1560 nm. Using ${\kappa }_{{{{{{{{\rm{t}}}}}}}}}^{0}$ and *β* from SMS, we can predict the total OAM cavity linewidth (*κ*_t_) and OAM ejection efficiency (*κ*_e_/*κ*_t_) through Eqs. (2) and (3), with only one free parameter *q*_0_.

In the top panel of Fig. 3c, we plot the measured *κ*_t_ for a series of OAM devices, where *N* has been varied so that *l* ranges between −165 and 0, and for three different values of *A*. We find that this experimental behavior agrees well with our model using the measured SMS values and *q*_0_ = 2, as shown by the different color solid curves in Fig. 3c. The width of the curves represents the uncertainty in the predictions due to the uncertainties of ${\kappa }_{{{{{{{{\rm{t}}}}}}}}}^{0}$ that come from nonlinear least-squares fits to the SMS transmission data (see the “Methods” section). We note that the predictions deviate from experiments near *l* = 0 for large *A*, with the inset zooming in on this behavior with adjacent *l* from −2 to +2. This low-radiation-loss mode only happens at *l* = 0, which has been used in integrated microrings for single-mode lasing^42^, and its physics is related to a bound state in the continuum phenomenon ^43,44^ induced by the photonic crystal structure.

The bottom panel of Fig. 3c shows the estimated extraction efficiency *κ*_e_/*κ*_t_ as a function of *l*, where *κ*_e_ is experimentally determined from the measured *κ*_t_ (from the OAM devices) and the measured ${\kappa }_{{{{{{{{\rm{t}}}}}}}}}^{0}$ from the SMS devices. The experimental data is again matched well by the model, particularly for larger values of *A*, where the model results are shown as solid curves whose widths are determined by the aforementioned uncertainties in the experimental SMS data. Importantly, the model contains no free parameter other than measured from experiments, except *q*_0_ = 2, which represents the upward and downward OAM emission paths. Between the two panels of Fig. 3c, we see the basic trend that the estimated OAM ejection efficiency and total cavity linewidth both increase in moving from *l* = −165 to *l* = 0. The measured upper bound of OAM ejection efficiency and total cavity linewidth also scale with modulation amplitude *A* as expected, with the level of agreement between theory and experiments improving with increasing *A*. The estimated ejection efficiency reaches *κ*_e_/*κ*_t_ = (80 ± 3)% at *l* = −105 and *A* = 16 nm, with *κ*_t_/(2*π*) of (1.19 ± 0.02) GHz and thus *Q*_t_ of (1.62 ± 0.02) × 10^5^. This efficiency is further increased to *κ*_e_/*κ*_t_ = (90 ± 1)% at *l* = −15, with a broadening of *κ*_t_ to (2.6 ± 0.2) GHz governed by Eq. (3).

We emphasize here that *κ*_e_/*κ*_t_ in Fig. 3c represents the upper bound of the OAM ejection efficiency, not the directly measured OAM ejection efficiency. In other words, any other coupling (i.e., loss) channels will contribute to the *κ*_e_ term and decrease the true OAM ejection efficiency. So far, we have not been able to either confirm or deny other contributions, for example, in-plane radiation to slab or surface modes, though our measurements bound how large they could be. In particular, the generally good agreement between our measured total loss rate and that predicted by Eq. (3) suggests that in the vast majority of cases (different modulation amplitude and *l* numbers), the coupling rate to any potential auxiliary channels is lower than the dominant loss channels we have focused on, that is, the intrinsic loss rate, waveguide coupling rate, and OAM ejection rate. Ultimately, direct experimental verification of the OAM efficiency would be quite valuable. However, in our current scheme, such verification is limited by many factors, including the high numerical aperture of the optics required to collect all of the emission for large *l*, the simultaneous presence of both CW and CCW (±*l*) emission, and the simultaneous emission in both the upwards (to air) and downwards (to the substrate) propagation directions.

A factor that degrades the data quality yet is difficult to count into error bars arises from the technical difficulty to identify and fit resonances in the regime of doublet splittings on par with intrinsic loss rates (i.e, a merged doublet) properly. This factor is particularly important when the OAM emission rate is small at large *l*, but becomes negligible when the OAM emission rate is high at larger *A*s and smaller *l*. Moreover, according to the fiber Bragg grating theory^45^, total internal reflection (i.e., in-plane momentum outside of the cladding light cone) is expected to turn off the OAM emission channel (*κ*_e_ = 0) for large *l*, which requires further investigation in our platform.

We also perform imaging of the OAM microring modes to confirm their spatial behavior as a function of *l*. As noted earlier, Fig. 2 shows the results for microring with *m* = 165 and *N* = 169. Rather than a pure *l* = −4 state, the images are consistent with the emission containing both *l* = −4 and *l* = 4 contributions, resulting in 4 × 2 antinodes in the measured distribution. Similar behavior has been observed in other OAM microcavity works^31^, where it was attributed to the ejection of light from a standing wave cavity mode. In our case, the ejection of both CW and CCW light could be due to surface roughness or waveguide facet reflection at the edge of the chip. The back-coupling rate of this reflection seems to be smaller than the total linewidth (unlike the SMS case), so a clear splitting of resonance is not observed in general. Next, Fig. 3d displays the imaged OAM microrings fields near the surface of the cavities for a variety of OAM states with increasing ∣*l*∣, as determined by analyzing the images and counting the number of anti-nodes. OAM states from ∣*l*∣ = 0 to ∣*l*∣ = 60 are clearly observed; the observation of even higher-order OAM is likely limited by the numerical aperture of our imaging system. We note that in these measurements, devices with ∣*l*∣ = 1–3 had an additional SMS modulation imprinted on the device pattern to ensure standing wave modes for better interference visibility; this method is discussed further in the next section. A comparison of devices with and without SMS is analyzed in Supplementary Information.

We compare our results against finite-difference time-domain simulations, with the simulation methods outlined in the Supplementary Information. Dipole excitation is used to excite standing-wave WGMs to have a beating pattern in the intensity for OAM. Figure 3e shows that the simulation results qualitatively agree with the observed patterns. Plotted here is the Poynting vector projected in the vertical direction, that is, ${{{{{{{{\bf{S}}}}}}}}}_{z}=({{{{{{{\bf{E}}}}}}}}\times {{{{{{{\bf{H}}}}}}}})\cdot \hat{{{{{{{{\bf{z}}}}}}}}}$, in the mid-field above the surface of the microring. The Supplementary Information provides further simulations of emitted OAM for both standing-wave and traveling-wave WGMs.

Finally, we emphasize that the observed *Q*s, in addition to following the predicted trends based on the SMS devices and Eqs. (1)–(3), are more than two orders of magnitude higher than those demonstrated in previous OAM generators based on microring resonators^27,31^, while simultaneously exhibiting a high estimated ejection efficiency. For example, the ∣*l*∣ = 60 mode has *Q*_t_ ≈ 5 × 10^5^ and an estimated ejection efficiency of 40% for *A* = 4 nm and *Q*_t_ ≈ 2 × 10^5^ and an estimated ejection efficiency of 65% for *A* = 8 nm. Such high-*Q*s are particularly promising for enhancing light–matter interactions, for example, to create Purcell-enhanced quantum light with OAM from a quantum emitter^31^, to realize coherent spin-photon interfaces^46^, or to mediate nonlinear wave mixing interactions such as Kerr comb generation and entangled-photon pair generation with the output fields encoded in OAM states^47^.

### Combining SMS and OAM coherently

**Fig. 4:**
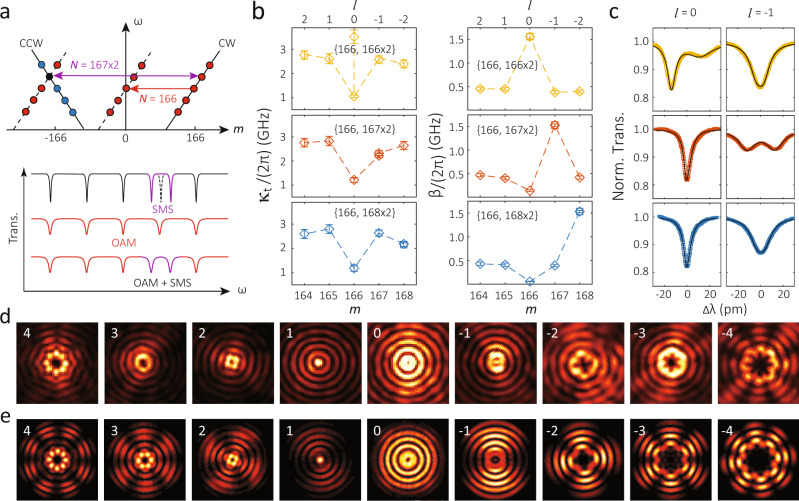
Coherent implementation of OAM and SMS simultaneously. **a** Schematic band diagram illustration (top) and expected transmission spectrum (bottom) when selective mode splitting (SMS) and OAM are coherently implemented together. **b** Here, we have three devices with the same OAM modulation of *N* = 166 and *A* = 16 nm but different SMS modulation of *N* = {166, 167, 168} × 2 and *A* = 2 nm in yellow, red, and blue, respectively. The left column displays the total dissipation rate from the cavity (*κ*_t_) while the right column displays the backscattering rate (*β*), both as a function of azimuthal mode order (*m*) and OAM state (*l*), with both obtained from nonlinear fits to transmission spectra for each mode (the error bars represent 95% confidence intervals of the nonlinear fits). Two data points are displaced in *κ*_t_ figures for the SMS modes, though overlapped within the error bars in the case of *m* = 167 × 2 and *m* = 168 × 2. **c** Normalized transmission spectra for the *l* = 0 and *l* = −1 modes (color) and their fitting curves (black) for each case, from which *κ*_t_ and *β* are extracted. **d** Images of the emitted light from the different *l* orders taken at a focus plane (70 ± 10) μm from the device. Different OAM states can be recognized by the distinctive `ripple' patterns generated. Here we show *l* from 4 to −4 as examples. **e** Predicted far-field patterns from three-dimensional finite-difference time-domain simulations. Here ∣**E**∣^2^ projected to the upper hemisphere with a radius of 1 m is plotted, see the Supplementary Information for details. While the OAM states are the same as in the experiments, a smaller device size (radius of 5 μm) and symmetric air cladding are used to keep the simulation size tractable.

So far we have been using a single-period grating for either SMS or OAM. Since both scattering processes are coherent, it is possible to combine them. For example, previous work has shown that combining multiple SMS periods through a multi-period grating (i.e. by simply adding up modulation with different *N*s) is practical and retains high cavity quality factors^39^. Here we use a dual-period grating to implement SMS and OAM together. For comparison, we study three cases with a fixed number of modulation periods for OAM at *N* = 166 and a varying number of modulation periods for SMS at *N* = 2 × {166, 167, 168}. In the band diagram displayed in the top panel in Fig. 4a, we illustrate the case in which the *m* = 166 modes are ejected to an *l* = 0 OAM state, and the *m* = ± 167 modes are coupled via SMS. The resulting cavity transmission is illustrated in the bottom panel, where SMS splits the *m* = 167 modes (in purple) without affecting linewidths and OAM broadens all the cavity linewidths (in red). Having both SMS and OAM should result in a coherent summation of both effects.

We examine the implementation of coherent OAM and SMS in three fabricated devices, as shown in Fig. 4b, c, where in contrast to the previous section, here we do not focus on a single azimuthal order modes, but instead examine a series of adjacent azimuthal order modes. Figure 4c shows representative transmission spectra (for *l* = 0 and *l* = −1, or equivalently *m* = 166 and *m* = 167). Figure 4b shows the extracted loaded cavity linewidths (*κ*_t_) created by OAM in the left column, and the right column shows the mode splitting (*β*) created by SMS. The overall behavior we observe is consistent with the expectation for coherent superposition of the OAM and SMS effects. The mode splittings are largest for the azimuthal mode targeted by the SMS modulation, while the OAM modulation is set to eject the *l* = 0 modes, and consistently shows a reduction in dissipation as observed in the previous section.

With or without SMS, our OAM devices always show standing-wave patterns in images taken both at the top surface of the microring, as shown in the previous section by Fig. 3d), and in the far-field, as we show in Fig. 4d. These standing-wave images resemble previous reports^31,46^, and is not an issue in many quantum systems, as the emitted light is intrinsically in both clockwise and counter-clockwise directions^31^. As noted earlier, their precise origin in our system requires further investigation. That being said, we find that the measured far-field images are in good agreement with the results of finite-difference time-domain simulations that incorporate a standing wave mode pattern, as shown in Fig. 4e.

Importantly, our results indicate that OAM emission does not have to lead to a mode splitting or a considerably broadened linewidth^27,32^, while the purity of OAM emission and its impact on the OAM efficiency (our estimate given by *κ*_e_/*κ*_t_ is an upper bound) require further investigation. For example, the popular square grating is effectively a composition of multiple frequency components, while only the fundamental frequency grating (as we employ with a sinusoidal modulation) is essential for OAM. The potential role that such multi-frequency components play in excess loss and backscattering is still an open question, and to this end, our approach from SMS to OAM can be extended to these structures to perform a quantitative evaluation.

## Discussion

We have demonstrated high-*Q* optical microcavities with controllable and efficient OAM ejection. By linking OAM ejection to the closely related effect of selective mode splitting (SMS) due to backscattering in a microresonator, we present a predictive model for the OAM cavity linewidth and ejection efficiency. We showcase twisted light with *l* from 0 to 60 and *p* = 0 (i.e., fundamental in the transverse direction), and it should be straightforward to extend to larger *l* and *p*. Our results will provide quantitative and analytical guidance in electromagnetic designs for applications including OAM multiplexing^9–12^, spatiotemporal shaping^34^ using frequency combs^37^, and quantum entanglement applications^13–15^. Future scientific understanding includes the origin of the coefficient *q*_0_ that relates the OAM ejection rate to the cavity finesse, azimuthal mode number, OAM state, and backscattering rate for the analogous SMS device. Additional studies to undertake include the effects of the light cone(s) defined by the cladding and substrate layers on *κ*_e_ and the origin of the apparent +*l* and −*l* superposition in the ejected light. Further important engineering tasks include using metamaterial structures with high numerical aperture to collect highly twisted OAM light, collecting/multiplexing OAM light into the optical fibers, and using OAM states to control/manipulate atomic states on top of the photonic chip.

## Methods

### Fabrication method

The stoichiometric Si_3_N_4_ layer is grown by low-pressure chemical vapor deposition with a nominal thickness of 500 nm on a SiO_2_ layer ≈3 μm thick and grown via thermal wet oxidation of a 100 mm diameter Si wafer. The Si_3_N_4_ layer thicknesses, as well as its wavelength-dependent refractive index, were confirmed using spectroscopic ellipsometry, with the index fitted to an extended Sellmeier model. A layer of positive-tone resists, Zep520A, ≈650 nm in thickness, is spun on top of the Si_3_N_4_ layer and exposed by a 100 keV electron-beam lithography system. The device layout is prepared using the Nanolithography Toolbox^48^, a free software package developed by the Center for Nanoscale Science and Technology at the National Institute of Standards and Technology. The pattern in use has a resolution of 1/8 nm for the in-plane grids and has an angular resolution of *π*/max(*N*)/12 for the inside modulation, where max(*N*) corresponds to the largest number of cell numbers (i.e., smallest period lengths) in use. During the lithography, the minimal grids are further increased to 2 nm due to the shot pitch limitation of the electron beam system in use for a 500 pA electron current. We can observe selective mode splitting down to a nominal modulation amplitude *A* = 1/8 nm using this method while maintaining a nearly linear dependence of mode splitting on amplitude. This observation is quite surprising considering the fracturing of the 2 nm shot pitch of the electron beam and its proximity effects, and it requires further study to clarify the underlying mechanism in terms of the actual pattern geometry relative to the designed one. Once the exposed pattern was developed, it was transferred to the Si_3_N_4_ using a CHF_3_:O_2_ reactive ion etch (RIE) chemistry with 30:5 standard cubic centimeters per minute (sccm) flow of each gas at a chamber pressure of 15 milliTorr (mTorr), with a rate of ≈30 nm per minute. The etching uses 150 W RIE power and has a DC bias voltage of 400 V. The remnant resists and deposited polymer during the etching process are chemically cleaned by Nano-Strip at 80 °C for 3 h. A SiO_2_ lift-off process is performed so that the microrings have a top air cladding while the input/output edge-coupler waveguides have a top SiO_2_ cladding. Such top and bottom SiO_2_ claddings create more symmetric modes for coupling to optical fibers, reducing the fiber-chip facet coupling loss to 2–3 dB per facet. The oxide lift-off process is based on photolithography, plasma-enhanced chemical vapor deposition of SiO_2_ with an inductively coupled plasma source, and the chemical removal of the photoresist. After the lift-off process, the chips are diced and polished and annealed at ≈1000 °C in an N_2_ environment for 4 h. Finally, we note that certain commercial products or names are identified to foster understanding. Such identification does not constitute a recommendation or endorsement by the National Institute of Standards and Technology, nor is it intended to imply that the products or names identified are necessarily the best available for the purpose.

## Supplementary information

Supplementary Information

## Data Availability

The data that supports the plots within this paper and other findings of this study are available from the corresponding authors upon request.
