# Supplementary material for: Highly-twisted states of light from a high quality factor photonic crystal ring
Source: Nat Commun. 2023 Feb 27;14:1119. doi: 10.1038/s41467-023-36589-8 (PMC9971168; doi:10.1038/s41467-023-36589-8)
Supplement: Supplementary file 1 — Supplementary Information [file 41467_2023_36589_MOESM1_ESM.pdf]

Supplementary Material
Highly-twisted states of light from a high quality factor photonic crystal ring

Xiyuan Lu,[1,2,*] Mingkang Wang,[1,3] Feng Zhou,[1,2] Mikkel Heuck,[4] Wenqi Zhu,[1] Vladimir A. Aksyuk,[1] Dirk R. Englund,[5] and Kartik Srinivasan[1,2,†]

[1]*Microsystems and Nanotechnology Division, Physical Measurement Laboratory, National Institute of Standards and Technology, Gaithersburg, Maryland 20899, USA*
[2]*Joint Quantum Institute, NIST/University of Maryland, College Park, Maryland 20742, USA*
[3]*Department of Chemistry and Biochemistry, University of Maryland, College Park, Maryland 20742, USA*
[4]*Department of Electrical and Photonics Engineering, Technical University of Denmark, Kgs. Lyngby 2800, Denmark*
[5]*Department of Electrical Engineering and Computer Science, Massachusetts Institute of Technology, Cambridge, Massachusetts 02139, USA*

(Dated: 2023-02-07)

In this supplementary material, we present a derivation of the equation linking SMS and OAM from the perspective of mode coupling, and the simulation method in use to calculate mid-field and far-field radiation patterns from the devices.

I. SUPPLEMENTARY NOTE 1: COUPLED-MODE EQUATIONS

In this section, we give coupled-mode equations linking OAM and SMS, leading to Equation (1) of the main text. First, the coupled mode equations for the clockwise ($\circlearrowright$) and counter-clockwise ($\circlearrowleft$) waves in the resonator can be given by the following:

$$\frac{d\tilde{A}_{\circlearrowright}}{dt} = (i\Delta\omega - \kappa_0/2)\ \tilde{A}_{\circlearrowright} + i\beta\ \tilde{A}_{\circlearrowleft} + i\sqrt{\kappa_{\rm c}}\tilde{S}_{\circlearrowright}, \tag{1}$$

$$\frac{d\tilde{A}_{\circlearrowleft}}{dt} = (i\Delta\omega - \kappa_0/2)\ \tilde{A}_{\circlearrowleft} - i\beta^*\ \tilde{A}_{\circlearrowright} + i\sqrt{\kappa_{\rm c}}\tilde{S}_{\circlearrowleft}. \tag{2}$$

The field amplitudes are normalized so that $U_{\circlearrowright} = |\tilde{A}_{\circlearrowright}|^2$ and $U_{\circlearrowleft} = |\tilde{A}_{\circlearrowleft}|^2$ represent the intracavity optical energy. The cavity detuning is given by $\Delta\omega = \omega - \omega_0$, where ω is the angular frequency and ω_0 is the central frequency of the cavity resonance. Only linear interaction and coupling terms are considered, and all nonlinear interaction terms are not included. We assume these two propagating waves have identical intrinsic loss rate (κ_0) and microring-waveguide coupling rate ($\kappa_{\rm c}$). In the selective mode splitting (SMS) case, the backscattering induced by the photonic crystal structures (β) can always be set to a real parameter, which is equivalent to defining the relevant phases of two travelling waves by the imprinted modulation pattern. The last terms are source terms, and $P_{\circlearrowright} = |\tilde{S}_{\circlearrowright}|^2$ and $P_{\circlearrowleft} = |\tilde{S}_{\circlearrowleft}|^2$ represent the clockwise and counter-clockwise input powers in the waveguide. This interaction ends up renormalizing two propagating waves into two standing waves, $\tilde{A}_\pm = (\tilde{A}_{\circlearrowright} \pm \tilde{A}_{\circlearrowleft})/\sqrt{2}$, given by:

$$\frac{d\tilde{A}_\pm}{dt} = [i(\Delta\omega \mp \beta) - \kappa_0/2]\ \tilde{A}_\pm + i\sqrt{\kappa_{\rm c}}\tilde{S}_\pm. \tag{3}$$

Here $\tilde{A}_\pm$ have the same cavity linewidth of $\kappa_{\rm t}^0 = \kappa_0 + \kappa_{\rm c}$, with the central resonance frequencies at $\omega_\pm = \omega_0 \pm \beta$, respectively. The source term follows a similar definition of $\tilde{S}_\pm = (\tilde{S}_{\circlearrowright} \pm \tilde{S}_{\circlearrowleft})/\sqrt{2}$.

From previous SMS works [1, 2], β can be calculated for an optical mode with shifting boundaries [3],

$$\beta = \frac{\omega_0}{2}\frac{\int dS\cdot A\left[(\epsilon_{\rm d} - \epsilon_{\rm c})|E_\parallel|^2 + (1/\epsilon_{\rm c} - 1/\epsilon_{\rm d})|D_\perp|^2\right]}{\int dV\epsilon(|E_\parallel|^2 + |E_\perp|^2)}, \tag{4}$$

where $E_\parallel$ ($D_\parallel$) and $E_\perp$ ($D_\perp$) are the electric field components (displacement field components) of the unperturbed optical mode that are parallel ($\parallel$) and perpendicular ($\perp$) to the modulation boundary dS, respectively. ϵ represents

*Electronic address: xiyuan.lu@nist.gov
†Electronic address: kartik.srinivasan@nist.gov

the dielectric function of the material, including the dielectric core ($\epsilon = \epsilon_\mathrm{d}$) and the substrate ($\epsilon = \epsilon_\mathrm{s}$) or cladding material ($\epsilon = \epsilon_\mathrm{c}$). Considering a photonic crystal microring with a fixed outer radius but modulated ring width of $W(\phi) = W_0 + \sum_n A_n \mathrm{cos}(n\phi)$, the standing-wave mode with a larger frequency has a dominant displacement field $D(\rho, \phi, z) = D(\rho, z)\mathrm{cos}(m\phi)$. Equation (4) can be written as:

$$\beta = \sum_n \frac{A_n \omega_0}{2} \frac{\int dS (1/\epsilon_\mathrm{c} - 1/\epsilon_\mathrm{d}) |D(\rho, z)|^2 \mathrm{cos}^2(m\phi)\mathrm{cos}(n\phi)}{\int dV \epsilon (|E_z|^2 + |E_\phi|^2 + |E_\rho|^2)\mathrm{cos}^2(m\phi)}. \tag{5}$$

This integral does not contain polar angle (θ), assuming the dominant electric field is in the radial direction and the sidewall of the modulation is straight. The azimuthal part can be integrated separately, so that:

$$\beta = \sum_n g A_n \int_0^{2\pi} d\phi \mathrm{cos}^2(m\phi)\mathrm{cos}(n\phi)/\pi = \sum_n g A_n (\delta_{n,0} + \delta_{n,2m}/2) = g_{mm}(A_0 + \frac{A_{2m}}{2}), \tag{6}$$

where $\delta_{i,j} = 1$ when $i = j$ and vanishes otherwise. g is defined as:

$$g \equiv \frac{\omega_0}{2} \frac{\int dS (1/\epsilon_\mathrm{c} - 1/\epsilon_\mathrm{d}) |D(\rho, z)|^2}{\int dV \epsilon (|E_z|^2 + |E_\phi|^2 + |E_\rho|^2)}. \tag{7}$$

While we derive the Eqs. (4-7) considering the shifting boundary for two standing-wave modes, this same parameter (β) is also the interaction term for two propagating waves described in Eqs. (1-2). In particular, Eq. (6) can be viewed as the interaction of clockwise (m) and counter-clockwise ($-m$) modes with the grating of (n) cells. The phase in the cos terms are set to zero here, which yields the maximal interaction/coupling rates, as the modes typically tend to maximize or minimize their energy. However, in specific cases the phase can be shifted, with one example being the phase shift for the bound standing-wave state at $l = 0$ OAM, as discussed in the main text.

In the OAM cases, for the travelling wave modes, the coupled mode equations are given by:

$$\frac{d\tilde{A}_{\circlearrowright}}{dt} = [i\Delta\omega - (\kappa_0/2 + \beta')] \tilde{A}_{\circlearrowright} + i\sqrt{\kappa_c}\tilde{S}_{\circlearrowright}, \tag{8}$$

$$\frac{d\tilde{A}_{\circlearrowleft}}{dt} = [i\Delta\omega - (\kappa_0/2 + \beta')] \tilde{A}_{\circlearrowleft} + i\sqrt{\kappa_c}\tilde{S}_{\circlearrowleft}. \tag{9}$$

There are no coupling of the clockwise and counter-clockwise modes induced by the grating. Instead, the grating introduces couplings from the WGM of azimuthal order m to an ejected free-space OAM mode $l = m - N$ for $m < N < 2m$ (and $-m$ to $-l$), and β' is the scattering rate. For the standing-wave modes, either by accidental mode splitting created by the random sidewall roughness [4], or another added grating for SMS, as demonstrated in the last section in the main text, the equation is given by:

$$\frac{d\tilde{A}_{\pm}}{dt} = [i\Delta\omega - (\kappa_0/2 + \beta')] \tilde{A}_{\pm} + i\sqrt{\kappa_c}\tilde{S}_{\pm}. \tag{10}$$

Here the β' term represents the scattering rate from a standing wave mode with $|m|$ to OAM modes with $|l|$ (consisting of $\pm l$ equally).

From Johnson et al.'s theory [3], the calculation of the coupling of two modes is identical to the energy shift to one mode itself. The SMS is a perfect example in that β can be considered as either the interaction of two travelling-wave modes, or the energy shift of a standing-wave mode from the modulated boundary. The OAM case is similar but more subtle: one can consider the OAM and WGM as being two modes, as the OAM is a radiating mode and the WGM is a confined mode. Yet, it is also not wrong to consider OAM and WGM to be two parts of the same mode in a broader definition, for the reason that the OAM mode is directly ejected out of the WGM mode, and they have the same mode profile before ejection (identical in polarization, linewidth, coupling, etc.). In both cases, if we follow Johnson et al.'s theory in a similar fashion, we have β' in the following:

$$\beta' = \frac{\omega_0}{2} \frac{\int dS \cdot A (1/\epsilon_\mathrm{c} - 1/\epsilon_\mathrm{d}) D_\perp D_\perp^e}{\int dV \epsilon (|E_\parallel|^2 + |E_\perp|^2)}, \tag{11}$$

The only difference between Eq. 11 and Eq. 4 is $D_\perp^e$ instead of $D_\perp$ (in both cases we assume that $E_\parallel$ is much smaller than the perpendicular field components). This term can be estimated from $D_\perp$ by considering three factors: (1) considering the ejected field has the same cross-sectional mode profile in the microring but without a cavity enhancement, we have $D^e = D/\sqrt{F_t/(2\pi)}$ since F_t is defined in terms of energy (hence the square root) and phase

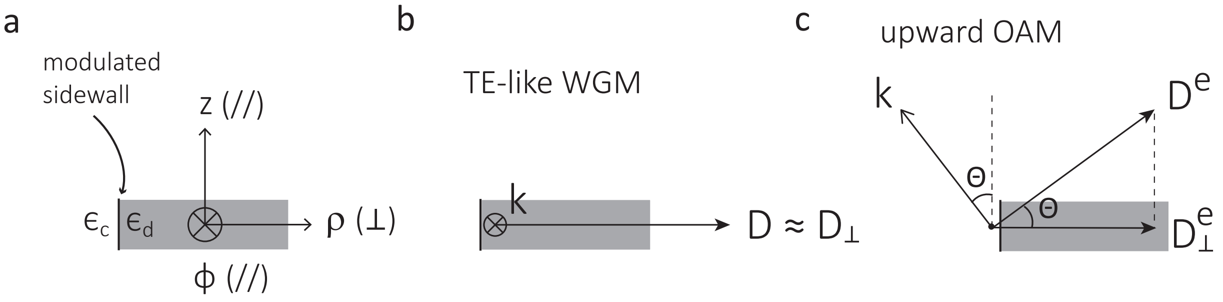

FIG. S1: **Schematic illustration of the WGM and OAM fields at the modulated sidewall.** **a** Cross-sectional drawing of the microring and the coordinates (ρ, ϕ, z) in use. The device layer and the cladding have dielectric constants of ϵ_d (in gray) and ϵ_c (in white), respectively. The modulated sidewall is emphasized by a black solid line. **b** For a transverse-electric-like (TE-like) WGM, the dominant electric displacement is in the radial direction, that is, perpendicular to the sidewall in modulation. **c** For the ejected OAM mode, the dominant field direction depends on the nominal ejection angle (θ) that is determined by angular momentum conservation. We illustrate upward OAM here, and downward OAM possesses mirror symmetry for this illustration.

(hence the 2π); (2) since we are considering transverse-electric-like WGMs whose dominant field components are in the radial direction, we have $D_\perp \approx D$, as illustrated in Fig. S1(a,b); and (3) the perpendicular projection can be estimated by the nominal ejection angle of the OAM beam from angular momentum conservation, as discussed in the main text, that is, $D^e_\perp = D^e \cos(\theta)$ and $\theta = (l/m)(\pi/2)$, as illustrated in Fig. S1(c). Considering these three factors, Eq. 11 can thus be written as

$$\beta' = \sum_n \frac{A_n\omega_0}{2} \frac{\int dS(1/\epsilon_\mathrm{c} - 1/\epsilon_\mathrm{d})|D(r,z)|^2 \cos(m\phi)\cos(l\phi)\cos(n\phi)}{\int dV \epsilon(|E_z|^2 + |E_\phi|^2 + |E_\rho|^2)\cos^2(m\phi)} \frac{q_0\cos(\theta)}{\sqrt{F_t/(2\pi)}}. \tag{12}$$

Here an additional term of q_0 is added in, considering that the emitted OAM light can be either upwards or downwards, which carries an additional factor of 2 compared to backward scattering in the SMS case. The value of this $q_0 = 2$ is validated in the experiments in the main text as a general trend, but its relationship to the light cone (and asymmetric cladding) requires further investigation. The azimuthal integral can be separated and yields similar results when $l = m - n$, so that Eq. 12 can be reduced to:

$$\beta' = \beta \frac{q_0 \cos(\theta)}{\sqrt{F_t/(2\pi)}}. \tag{13}$$

Considering the role of β' in Eqs. (8-10), we have Eq. (1) in the main text,

$$\kappa_e = 2\beta' = q_0 \frac{2\beta}{\sqrt{F_t/(2\pi)}} \cos(\theta). \tag{14}$$

Though we are considering standing-wave fields here as examples, the ejection rate (κ_e) is the same for travelling waves, similar to the coupling rate (β) that is used both in standing-wave and traveling-wave cases for SMS. Also, while our derivation is for transverse-electric-like fields, transverse-magnetic-like fields have the same results of Eq. 14, though the major contribution of β and κ_e is contributed from the parallel field components.

II. SUPPLEMENTARY NOTE 2: SIMULATION METHOD

In this section, we discuss the method to simulate the mid-field and far-field images used in the main text. We employ finite-difference-time-domain (FDTD) simulations using the commercially available software Lumerical. We note that in this paper, certain commercial products or names have been identified to foster understanding. Such identification does not constitute recommendation or endorsement by the National Institute of Standards and Technology, nor is it intended to imply that the products or names identified are necessarily the best available for the purpose.

To reduce the required simulation resources, we consider smaller ring radii of 5 μm and 12 μm as well as a modulation amplitude of $A = 100$ nm. We consider devices in three geometries. The first device, shown in Fig. S2(a), consists of an isolated ring with air cladding on the top and bottom. The second device in Fig. S2(b) has an additional bus

waveguide below the ring and a 3 μm thick SiO_2 bottom cladding. The third device in Fig. S2(c) has a bus waveguide below the ring, SiO_2 bottom cladding, and a Si substrate. The bus waveguide is 750 nm wide and separated from the ring by a gap of 350 nm. We use the refractive indices of $n_{\rm SiN} = 2$, $n_{\rm SiO_2} = 1.45$, and $n_{\rm Si} = 3.48$. The grid-spacing used is 30 nm in all three directions (note that we use Lumerical's ability to automatically set a coarser grid away from the ring).

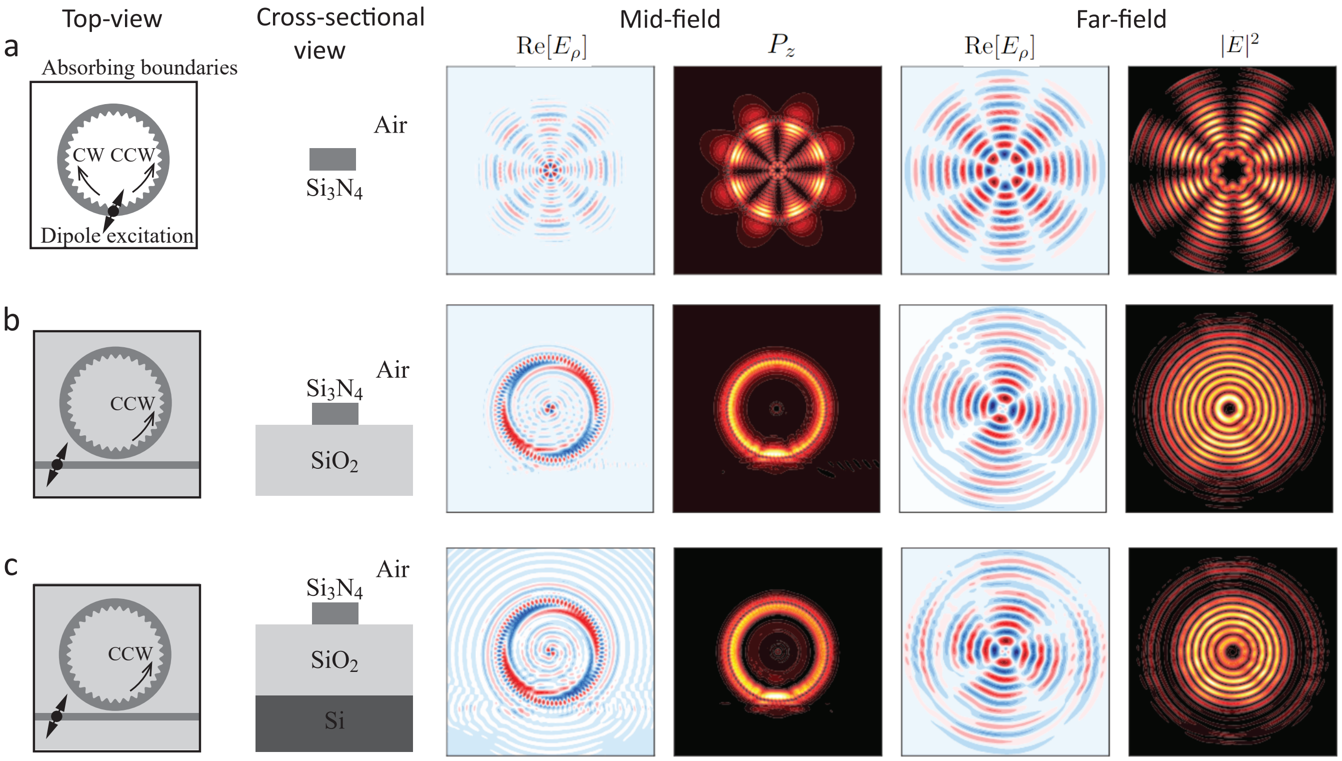

FIG. S2: **Examples of cavity modes calculated by FDTD simulations with three different simulation schemes. a** Isolated ring with dipole excitation. **b** Bus waveguide-coupled ring with 3 μm SiO_2 bottom cladding. **c** Bus waveguide-coupled ring with 3 μm thick SiO_2 bottom cladding and Si substrate. The four columns of images plot $|E|^2$, midfield: $\mathrm{Re}[E_\rho]$, P_z, far field: $\mathrm{Re}[E_\rho]$, $|E|^2$, respectively.

Figure S2(a) shows an example of the isolated ring with $N = 78$, which results in a resonant mode at $\lambda = 1677$ nm with $m = 74$, corresponding to $l = -4$. The modes are excited using 10 dipole sources evenly distributed over one period of the waveguide modulation inside the ring. Several dipoles are used to ensure that all longitudinal modes are excited. The mid-field profile plotted in Fig. S2(a) is calculated as a running Fourier transform of the time-dependent field amplitude, $\mathbf{E}(t)$, in the plane at $z = 0$. Note that Lumerical offers a setting to multiply the field amplitude by a ramping-function that eliminates any contribution from the sources. The dipole sources excite both clockwise and counterclockwise modes and the resulting mode shows an interference pattern between the two. The number of anti-nodes in $|\mathbf{E}|^2$ is therefore $2|l|$. In the mid-field images, $\mathrm{Re}[E_\rho]$ and P_z are plotted in a plane 1 μm above the waveguide surface, and in the far-field images, $\mathrm{Re}[E_\rho]$ and $|E|^2$ of the far field are calculated using Lumerical's built-in far field transformation routine. It is the field recorded 1 μm above the waveguide surface that is used in the transformation. Besides dipole excitation, standing-wave WGMs can be created by SMS (as described in the main text), random sidewall backscattering from the microring, and the chip facet reflection. The latter two typically lead to partial reflection instead of an equal contribution of clockwise and counter-clockwise modes, and will lead to more blurry (i.e., less visible) interference patterns.

Figure S2(b, c) shows mode profiles of structures including a bus waveguide with symmetric air cladding and with SiO_2 bottom cladding. Figure S2(c) also has a Si substrate underneath the SiO_2 bottom cladding. For both structures, the source of the FDTD simulation is an eigenmode of the bus waveguide propagating from left to right and exciting only the counterclockwise ring mode. The mode profiles are again only monitored at times after the source has died out and the fact that only the counterclockwise mode is excited is verified by light only leaking out to the right in the bus waveguide. We can clearly see that, with such travelling-wave excitation, the field does not show $2|l|$ beating patterns in either mid-field P_z or far-field $|E|^2$. In other words, the phase is varied by $l = -4$, but in intensity it is

uniform.

In particular, Fig. S2(c) suggests that the substrate reflection, that is, at the SiO_2/Si interface, cannot be used to explain the observed $2|l|$ beating pattern, in contrast to the facet reflection as shown in Fig. S2(a). Because of the symmetry, the device in simulation has $l = -4$ for the upward OAM (into air) and $l = 4$ for the downward OAM (into SiO_2). The $l = 4$ light is reflected at the SiO_2/Si interface to $l = -4$. Such reflected light with $l = -4$ can shift the phase of the original $l = -4$ light, but cannot create a beating pattern in intensity.

III. SUPPLEMENTARY NOTE 3: EXTRACTING κ AND β FROM TRANSMISSION SPECTRA.

We show here examples of extracting κ and β from representative transmission spectra for selective mode splitting (SMS) and orbital angular momentum (OAM) devices, more specifically, six devices with l = {-165, -60} and A = {4, 8, 16} nm in Fig. 3(c) in the main text. In Fig. S3(a-c), SMS devices show increased mode splitting in the targeted mode when A increases, but the overall optical quality factor (Q_t) is not strongly affected (i.e., remains within a common range of Q_t values determined by the uncertainties in the nonlinear least squares fits to the transmission data). In Fig. S3(d-f), OAM devices have decreased Q_t and broader linewidths ($\kappa_t = \omega/Q_t$, which is used throughout the main text) when A increases. The cavity transmission becomes under-coupled (shallow in dip) from the perspective of waveguide coupling, as the microring waveguide coupling rate (κ_c) stays the same while the OAM ejection rate (κ_e) increases.

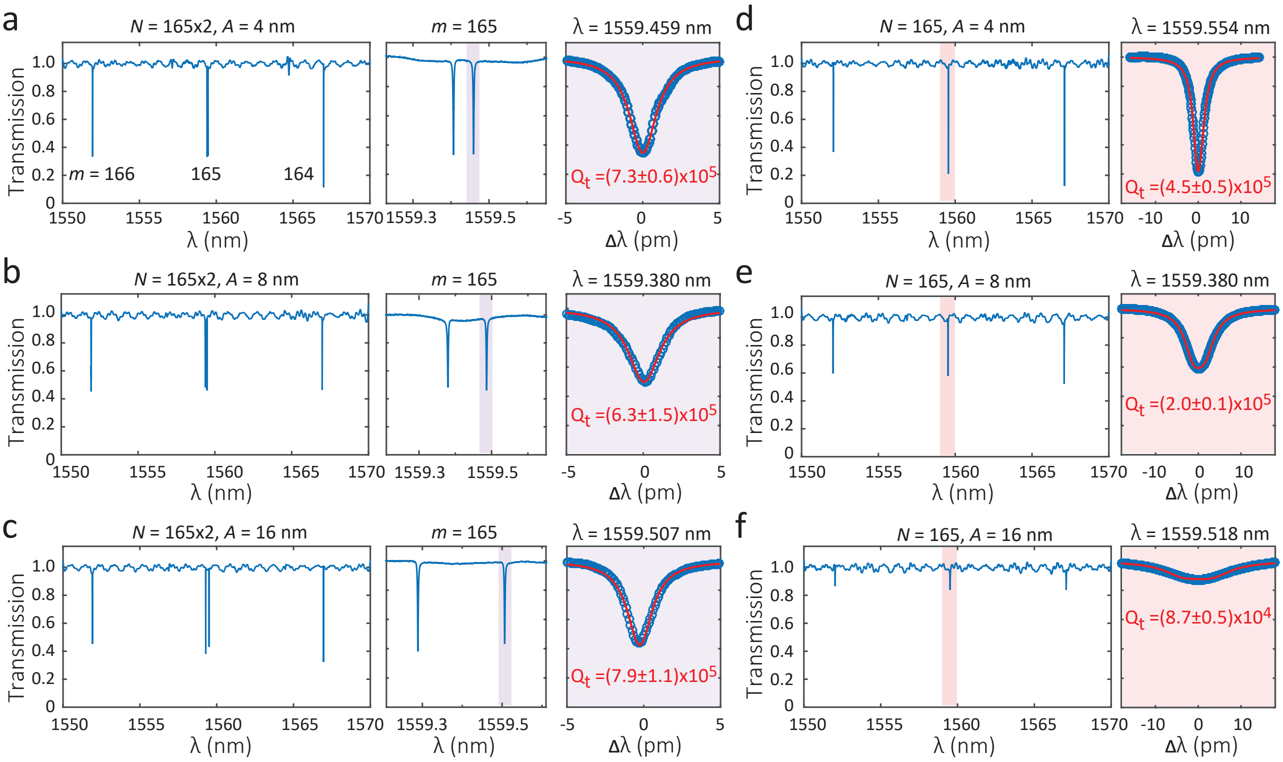

FIG. S3: **Examples of characterization of selective mode splitting (SMS) and orbital angular momentum (OAM) devices.** **a-c** Cavity transmission of the SMS devices with $N = 165{\times}2$ and A from 4 nm to 16 nm. The splitting increases when A increases. **d-f** Cavity transmission of the OAM devices with $N = 165$ and A from 4 nm to 16 nm. The linewidth increases when A increases. The uncertainty in Q_t is given by a 95 % confidence range of nonlinear fitting.

IV. SUPPLEMENTARY NOTE 4: DECREASED OAM EMISSION NEAR $l = 0$ WITH LARGE A

In the inset of Fig. 3(c) in the main text, a decrease of OAM emission is observed near $l = 0$ with only large A. Such a decrease seems abnormal when compared to other sets of data with smaller As, and does not agree with the equation we proposed (by roughly a factor of 2). In this section, we focus on discussing this abnormal behavior.

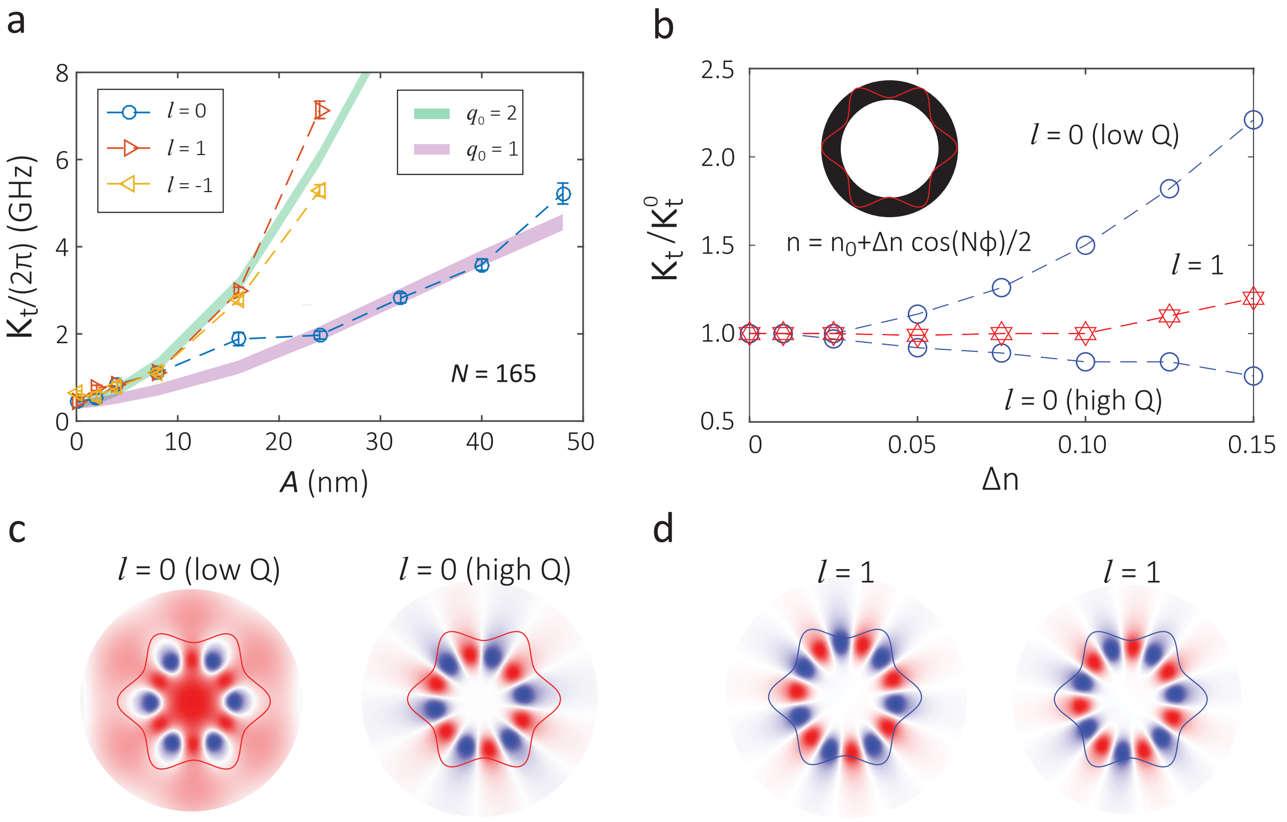

FIG. S4: **Understanding the abnormal behavior at $l = 0$ with large A.** **a** Experimental study of κ_t at $l = 0$ for A from 0 nm to 48 nm. **b** A two-dimensional finite-element-method simulation of normalized dissipation rate for l=1 and l=0 modes, with index modulated instead of the ring width. The dissipation rate (κ_t) is normalized to the dissipation rate of a control microring (κ_t^0) without index modulation ($n = n_0$). For a photonic crystal ring with index modulation, the refractive index is a function of azimuthal angle ϕ given by the inset equation, $n = n_0 + \Delta n cos(N\phi)/2$, where $n_0 = 3.5$ and the modulation period N is 6. For $m = 6$ modes, this device has two $l = 0$ standing-wave modes with different radiation rates. For $m = 7$ modes, this device has two degenerate $l = 1$ modes with the same radiation rates. **c** Numerical simulation of E_r for a WGM with $N = m = 6$ (l=0). The topology of the PhCR breaks the degeneracy in this case for two standing-wave modes, the left one with its field antinodes sitting at the edge with minimal index contrast, the right one with its field antinodes sitting at the the top and bottom of the grating seeing the maximal index contrast. **d** Numerical simulation of the electric field distribution of a WGM with $m = 7$ in a $N = 6$ (l=1) photonic crystal microring. These two modes have the same dissipation rates.

We first verify this behavior experimentally by measuring many devices with modulation amplitude A of 0 nm to 48 nm, as shown in Fig. S4(a), and focusing on the singular behavior of the dissipation κ_t at $l = 0$ for these devices. Compared to the closest two adjacent modes with $l = \pm 1$, it presents distinguishable lower dissipation rates when A is increased above 16 nm. A recent paper [5] has reported a decrease of radiation loss from the one-dimensional waveguide grating in silicon nanophotonics, where a state with reduced radiation is induced by the counter-interference of two radiation channels that are out of phase. Distinctively, for this mode with lower radiation loss, the light has its field center sitting at the edge of the square grating, instead of the top or bottom of the waveguide grating. Though a quantitative reduction of the radiation measured in Fig. S4(a) can be difficult to examine in simulation, we present here a simplified two-dimensional model in Fig. S4(b), using finite-element-method simulation to confirm we have a similar physics from the microring grating. In particular, using a microring with an index grating of N periods, we show that the l=0 modes have a splitting in their dissipation rate (given here in normalized units), with a higher-Q mode that shows a lower dissipation rate than the adjacent l=1 mode, and a lower-Q mode that shows a higher dissipation rate than the adjacent l=1 mode, for all values of the index modulation strength. In Fig. S4(c)-(d), we show the electric field distribution of WGMs for the l=0 and l=1 cases. For the l=1 case ($m = 7$ in a $N = 6$ photonic crystal microring) in Fig. S4(d), the two eigenmodes correspond to the standing waves mixed from the degenerate clockwise and counterclockwise traveling wave modes. As the electric field of each mode interacts with the dielectric structure in the same way (i.e., in terms of the position of the field antinodes and nodes with respect to the dielectric

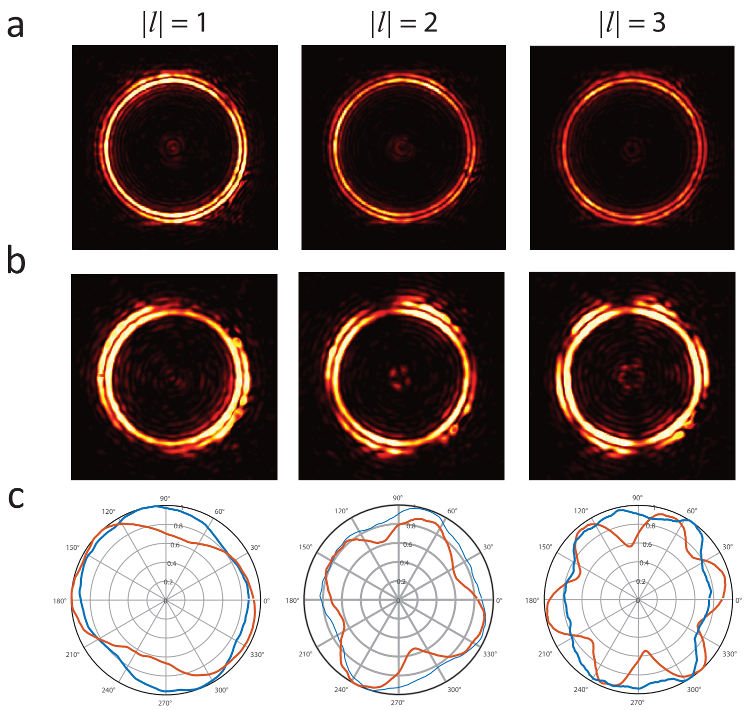

FIG. S5: **Infrared images for the devices without and with SMS.** **a** Device without SMS does not show clear visibility of the beating patterns. Here the mode has $m = 162$, and three devices have $N = \{163, 164, 165\}$, respectively. **b** Device with SMS showing clear beating patterns. Besides the OAM modulation in (a), an additional modulation with $N = 2 \times 162$ is added to couple and split the traveling wave modes into standing-wave modes. **c** Normalized intensity distribution as a function of azimuthal angle (ϕ) for the devices without (blue) and with (red) SMS. The devices with SMS have a better visibility in the $2|l|$ beating pattern.

modulation), without imperfections, they have the same frequency and the same loss rate. However, for a numerical simulation of E_r for a WGM with $N = m = 6$ (l=0 modes in Fig. S4(c)), the topology of the PhCR breaks the degeneracy, with the field antinodes for the two modes no longer equivalently sampling the dielectric, and gives rise to two standing-wave modes with different radiation loss.

We note that this phenomenon has been observed before in the photonic crystal ring [6]. That work achieves single-mode lasing by two factors: (1) the modulation amplitude is set to be quite large so all other modes except $l = 0$ have very low optical quality factors that cannot be measured in a transmission spectrum, and (2) the gain material preferentially selects the $l = 0$ mode (the only mode left with observable Q). Though the parameter space is quite different in that work, the fundamental principle seems to be the same, that is, the $l = 0$ mode has a decreased radiation compared to other modes when the modulation is large.

V. SUPPLEMENTARY NOTE 5: COMPARING OAM DEVICES WITH AND WITHOUT SMS.

We compare OAM devices with and without SMS for $|l| = 1$ to 3 in Fig. S5 . The OAM device without SMS has worse visibility in intensity beating, as the counter-clockwise WGM is likely from the back reflection of the pump laser, either from the chip facet or from the microring, and is thus much smaller in amplitude than the clockwise WGM. The method of implementing SMS and OAM has been discussed in the main text. The OAM device with SMS has standing-wave WGMs (equal clockwise and counter-clockwise WGMs) and hence nearly equal $+l$ and $-l$ OAM, resulting in more pronounced interference patterns.

VI. SUPPLEMENTARY REFERENCES

[1] Lu, X., Rogers, S., Jiang, W. C. & Lin, Q. Selective engineering of cavity resonance for frequency matching in optical parametric processes. *Appl. Phys. Lett.* **105**, 151104 (2014).

[2] Lu, X., Rao, A., Moille, G., Westly, D. A. & Srinivasan, K. Universal frequency engineering for microcavity nonlinear optics: multiple selective mode splitting of whispering-gallery resonances. *Photon. Res.* **8**, 1676–1686 (2020).

[3] Johnson, S. G. *et al.* Perturbation theory for Maxwell's equations with shifting material boundaries. *Phys. Rev. E* **65**, 066611 (2002).

[4] Weiss, D. S. *et al.* Splitting of high-Q Mie modes induced by light backscattering in silica microspheres. *Opt. Lett.* **20**, 1835–1837 (1995).

[5] Yulaev, A. *et al.* Exceptional points in lossy media enable decay-free wave propagation. *Nat. Nanotech.* **17**, 583–589 (2022).

[6] Arbabi, A., Kamali, S. M., Arbabi, E., Griffin, B. G. & Goddard, L. L. Grating integrated single mode microring laser. *Opt. Express* **23**, 5335–5347 (2015).
